# Correlates of long-acting reversible contraception uptake among rural women in Guatemala

**DOI:** 10.1371/journal.pone.0199536

**Published:** 2018-06-27

**Authors:** Kirsten Austad, Pooja Shah, Peter Rohloff

**Affiliations:** 1 Division of Women’s Health, Department of Medicine, Brigham and Women’s Hospital, Boston, Massachusetts, United States of America; 2 Wuqu’ Kawoq | Maya Health Alliance, Santiago Sacatepéquez, Guatemala; 3 UCSF School of Medicine, San Francisco, California, United States of America; 4 Division of Global Health Equity, Brigham and Women’s Hospital, Boston, Massachusetts, United States of America; University of North Carolina at Chapel Hill, UNITED STATES

## Abstract

**Objective:**

In many low-resource settings around the world utilization of long-acting reversible contraception (LARC) is low, in part due to access barriers. We sought to explore LARC utilization patterns as well as factors associated with LARC initiation by women seeking contraception in rural Guatemala from a program working to reduce contraception access barriers.

**Study design:**

We analyzed data from a program that provides family planning in six remote, primarily indigenous, villages in Guatemala with limited access to alternative health services. Methods are free and delivered directly within villages by culturally competent providers. We conducted a retrospective chart review of all 288 women who initiated a contraceptive method over a 16-month period and conducted a logistic regression to obtain adjusted odds ratios (OR) for predictors of LARC uptake.

**Results:**

Overall 79.2% of women elected a LARC method. More than half of women (49.8%) switched to LARC from short-acting hormonal methods. In the univariate analysis prior use of short-acting method (p = 0.014), number of prior methods (p = 0.049), and current contraceptive use (p<0.01) were significantly associated with choosing a LARC. In the logistic regression model current use of contraception remained significant (OR 3.29, 95% CI 1.67–8.04). Report of abnormal bleeding or other side effects from prior short-acting method use did not predict LARC uptake (p = 0.82 and p = 0.079).

**Conclusions:**

Most women in this marginalized population opted for a LARC method.

**Implications:**

Low utilization of LARCs may be related to service delivery factors. Further research is needed to validate these conclusions prospectively and in less selected populations.

## Background

An estimated 867 million women from low- and middle-income countries (LMICs) wish to avoid pregnancy [1]. Among those who reside in Central America, gains in family planning use over the past 20 years is largely due to sterilization and use of short-acting hormonal contraception (SAHC), such as injectables and combined oral contraceptives (COCs) [2, 3]. Long-acting reversible contraception (LARCs), including intrauterine devices (IUDs) and sub-dermal implants, offer a safe and cost-effective alternative that could help women space or limit births [2, 4, 5]. While their use is slowly rising in some developed nations [6], they remain uncommon in most LMICs [1, 2, 7, 8].

Given the complexity of contraceptive decision making, a large body of research informs the question of why LARCs are underutilized in LMICs. There is a general consensus that lack of knowledge regarding LARCs—linked with poverty and less access to formal education—is an important and prevalent cause of low LARC use [9–12]. In addition, demographic attributes of users, including younger age [11], and future fertility plans, such shorter interval to next desired pregnancy [13], are also associated with lower LARC uptake in previously published studies. Qualitative work has also documented pervasive myths about IUDs and implants among both patients [7, 14] and medical providers [15]. These—along with the side effect profiles and changes in menstruation inherent to the methods—may lead family planning clients to simply prefer injectables and COCs over implants and IUDs despite their increased efficacy.

At the same time, however, some evidence also supports the idea that the popularity of SAHC over LARC methods is more due to access barriers than women’s preferences. For example, mobile outreach has been a more effective strategy for LARC promotion than clinic-based provision [16], suggesting that convenience plays a role in demand. Furthermore, supply-side deficits including lack of trained providers to offer LARC methods and supply chain problems are common in LMICs and create supply side barriers for LARC access [17, 18]. Even when methods are theoretically available, evidence from the United States suggests that patient interest in LARCs is dependent on adequate counseling [19] and training of providers [20, 21]. Cost is another access barrier to contraceptive use that is likely to disproportionately decrease interest LARC methods relative to SAHC [22]. Lastly, other aspects of the service delivery environment—such as provider bias or distrust in services [2], which commonly impact adolescents, and general concerns over quality of care [23]—may also influence LARC use. Indeed, a demonstration project in Ethiopia to create youth friendly family planning services found that it successfully increased LARC uptake among the target population [24].

Given this complexity, it is unclear to what extent the lower demand for LARCs observed in most large LMIC surveys would be fixed simply by improving access and to what extent they reflect women’s true underlying preferences. This study seeks to inform this question in Guatemala, where the majority of need is filled by injectables and less than 4% of women use a LARC method [25]. Within Guatemala important differences exist between the approximately 40% of the population who identify as indigenous (primarily of Maya descent and who live in rural areas) and the majority population of European or mixed ancestry [25]. Maya women are nearly twice as likely to report an unmet need for contraception than women of European or mixed ancestry and are more likely to fill that need with short-acting methods instead of LARCs [25]. It remains unknown to what extent LARC acceptance would rise in Guatemala if access barriers to remote indigenous communities were removed.

To this end, we present data from a program to deliver family planning services to rural Maya women in the Guatemalan highlands. This project, implemented by Maya Health Alliance (MHA), is designed to overcome the access barriers Maya women face in in receiving health services through public clinics, including rural isolation, high out-of-pocket costs, language incongruence, and perceived low-quality of care in public facilities [26]. MHA offers contraception education through group classes, one-on-one counseling with mothers enrolled in the childhood malnutrition programs, and medical consultations of women presenting for preventive women’s health services (prenatal care, cervical cancer screening) or acute complaints. Indigenous nurses fluent in Mayan languages deliver all contraceptive methods—including COCs, injectables, sub-dermal implant, and IUDs—at no cost via mobile clinics and home visits scheduled on demand.

In this context, we examined rates of LARC and SAHC initiation in a retrospective cohort of rural Guatemala women seeking contraception from MHA. We hypothesized that, given the removal of many access barriers by the program, a preference for LARC initiation over SAHC methods would be observed. We also examined the relationship between demographic, reproductive and contraceptive use histories and LARC initiation to identify other correlates of LARC preference (over SAHC) in this setting of reduced access barriers.

## Materials and methods

### Program description

This study was a retrospective review of existing electronic medical records (EMR) data from Wuqu’ Kawoq | Maya Health Alliance (MHA), an NGO that provides comprehensive primary health care in Guatemala. Six small villages in central Guatemala receive contraceptive services from MHA and are represented in this analysis; as we have previously described, residents are majority indigenous Maya and, because of their rural location, have limited utilization of public clinics [27, 28].

MHA nurses document provision of contraception using a standardized form within the EMR, which includes menu-based multiple-choice responses to guide them through documenting patients’ gynecologic/obstetric history, current symptoms, physical exam, and clinical plan. Women can choose from point-of-care initiation of SAHC, including three-month injectable medroxyprogesterone acetate (DMPA or “injectable”) and COCs or LARC, including 4- or 5-year sub-dermal levonorgestrel implants and copper IUD (TCu380A). Condoms are offered as adjuncts and counseling on natural methods (such as lactational amenorrhea) is provided on request. Sterilization is not offered. Nurses use standardized clinical protocols to determine medical eligibility for each method, similar to existing and widely used quick start algorithms [29]. For women in whom pregnancy cannot be excluded but who desire a LARC, SAHC is offered as a bridge (as emergency contraception is not readily available in Guatemala) and pregnancy test is repeated in 2–3 weeks.

### Study design and sampling

Two authors (KA and PS) conducted an EMR search by date and type of form to identify all patients who received a contraceptive method from February 2015 (when the EMR form was first implemented) through October 2016. Full inclusion criteria for the analysis were: receipt of a contraceptive method within the study timeframe, female, age 18 or older, pre-menopausal, and at least one lifetime sexual partner. We manually extracted data from intake forms into a spreadsheet (PS) with the first 5% reviewed (by KA) to verify accuracy of the process.

We classified women as SAHC or LARC users based on the method being used at the end of the first visit during the study timeframe, unless a SAHC was given as a bridge to a LARC method (in which case she was classified as a LARC user). To allow for education of existing SAHC users, we classified patients who switched to a LARC during the first 3 month of project roll-out (to allow existing patients using SAHC the opportunity to switch) as LARC users. Subsequent encounters during which women switched or discontinued methods will be reported on in a future analysis.

The Institutional Review Boards of Partners Healthcare and MHA approved the study. Both IRBs waived the requirement for informed consent.

### Data analysis

We completed all analyses in Stata 14 (StataCorp Texas, USA). We summarized continuous variables—including age, number of pregnancies, number of living children, age at first pregnancy, and time since last pregnancy—using median and interquartile range (IQR) given their nonparametric distribution. Categorical variables are presented as frequencies according to column totals. We then used logistic regression with the binary outcome of LARC uptake (versus SAHC) to look for associations with each of these independent variables to produce unadjusted odds ratio and p-values. To further explore the impact of prior experiences with DMPA and COC use on method choice we also conducted a nested univariate analysis, using these same methods, for the sub-population of women who had previously used SAHC.

Next, multiple logistic regression was used to examine demographic and reproductive factors associated with choosing a LARC. For the model building process our overall aim was to include the fewest number of parameters required to identify important factors associated with LARC initiation over SAHC. We began by with age and number of pregnancies, factors known to influence contraception decision-making [11, 12]. Next, we sequentially incorporated all variables in Table 1, examining model fit parameters and variable inflation factors to monitor for collinearity. We report the model with the best fit, including OR, 95% CI, and indicators of model strength (Hosmer-Lemeshow goodness of fit and likelihood ratio tests).[30] In all models the variable representing number of prior methods was included as a categorical predictor (no method, 1 method, or 2 or more methods).

**Table 1:** Characteristics of overall study cohort and comparison of LARC and SAHC groups.

Variable		N	Total Population n = 288	Received LARC n = 228	Received SAHC n = 60
Age in years (median, IQR)			27.1 (22.8–32.5)	27.0 (22.7–31.8)	28.0 (23.5–34.0)
	Under 20	22	8%	7%	10%
	20 to 24	95	33%	33%	32%
	25 to 29	70	24%	26%	17%
	30 to 34	55	19%	18%	22%
	35 and over	46	15%	15%	20%
Pregnancies (median, IQR)			3 (2–4)	3 (2–4)	3 (2–4)
	0	1	< 1%	0%	2%
	1 or 2	131	46%	47%	40%
	3 or 4	92	32%	30%	43%
	5 or more	64	22%	24.%	15%
Number of living children (median, IQR)			2 (2–4)	2 (2–4)	2 (2–3)
	0	1	< 1%	0%	2%
	1 or 2	146	51%	53%	43%
	3 or more	141	49%	41%	55%
Age at first pregnancy in years (median, IQR)			19 (17–20)	19 (17–20)	19 (17–21)
	Under 15	7	3%	3%	2%
	15 to 19	165	61%	62%	59%
	20 to 24	76	28%	29%	27%
	25 or older	22	8%	7%	13%
Time since last pregnancy in months (median, IQR)			12 (6–24)	13 (13–24)	12 (7–24)
	Less than 6	71	26%	26%	25%
	6 to 24	139	50%	50%	51%
	More than 24	68	25%	24%	25%
> 1 lifetime sexual partners		273	14%	15%	14%
History of sexual or domestic violence		276	12%	11%	16%
Prior cervical cancer screening		288	47.6% (137)	47.4% (108)	48.3% (29)
Medical conditions limiting contraceptive use†		280	5.4% (15)	5.3% (12)	5.7% (3)
Previous SAHC use*		288	71.2% (205)	74.5% (170)	58.3% (35)
Previous LARC use		288	5% (14)	4.8% (11)	5.0% (3)
Previous natural method use		288	1% (3)	0.4% (1)	3.3% (2)
Number of prior contraceptive methods used in lifetime*					
	0	72	25% (72)	21% (48)	40% (24)
	1	165	57% (165)	62% (141)	40% (24)
	2 or more	51	18% (51)	17% (39)	19% (12)
Current use of SAHC or LARC at start of index visit*		288	50% (144)	55% (126)	30% (18)

Asterisk (*) denotes statistically significant difference between those who initiated LARC versus SAHC at p < 0.10.

†Includes hypertension, migraine, diabetes mellitus, and rheumatologic disease.

*p< 0.10 according to univariate analysis, thus included in multivariate logistic regression model.

## Results

### Characteristics of the sample

Chart review yielded 288 women who met inclusion criteria. The median age and number of pregnancies for the cohort was 27.1 years (IQR 22.8–32.5) and 3 pregnancies (IQR 2–4), with most women (66.2%) becoming pregnant for the first time in adolescence. Most users chose a LARC (79.2%) with a strong preference for implants (97.8%) over copper IUD (2.2%). Most who elected a SAHC preferred DMPA (96.7%), consistent with national trends (6). Overall 75.0% of women had used contraception in the past, but only 4.9% were prior LARC users. Additional clinical and demographic characteristics of the sample are detailed in Table 1.

The majority of individuals who chose implants directly transitioned from SAHC (49.8%), while those who chose the copper IUD were split equally among postpartum (40.0%) and former IUD users (40.0%). Never users comprised a greater percentage of those who elected DMPA than any other methods.

### Comparison of women who initiated LARC versus SAHC

In univariate analysis, there was no difference according to most demographic or clinical characteristics between users who elected LARC versus SAHC (Table 2). However, uptake patterns significantly favored LARC use for women using contraception at the beginning of the index visit (p<0.001) and those who previously used SAHC (p = 0.014). There was no significant difference between LARC and SAHC uptake for prior LARC (p = 0.76) or natural method (p = 0.11) users. Among women who chose a LARC, 78.9% had used at least one prior contraceptive method, whereas among women who chose SAHC, 60.0% had used at least one prior contraceptive method (p = 0.049).

**Table 2:** Results of logistic regression model for LARC uptake.

Variable	Unadjusted Odds Ratio (95% CI)	p-value	Adjusted Odds Ratio (95% CI)	p-value
Age	0.97 (0.93–1.02)	0.227	0.92 (0.85–0.98) *	0.013
Number of pregnancies	1.02 (0.89–1.16)	0.81	1.23 (1.00–1.51)	0.050
Number of living children	0.99 (0.86–1.15)	0.90		
Age at first pregnancy	0.95 (0.87–1.05)	0.35		
Time since last pregnancy	1.00 (0.99–1.01)	0.91		
>1 lifetime sexual partner	1.45 (0.59–1.86)	0.43		
History of sexual or domestic violence	0.61 (0.25–1.44)	0.26		
Prior cervical cancer screening	0.95 (0.53–1.69)	0.86		
Previous SAHC use	2.25 (1.23–4.09) *	0.014	1.51 (0.51–4.47)	0.46
Previous LARC use	1.27 (0.27–5.97)	0.76		
Previous natural method	0.12 (0.01–1.37)	0.11		
Number of prior methods used	1.58 (0.99–2.49)*	0.049	0.83 (0.36–1.91)	0.66
Current use of contraception	3.29 (1.75–6.20) *	<0.01	3.67 (1.67–8.04) *	< 0.01

Significant findings at p < 0.05 are indicated with an “*”.

CI = confidence interval.

### Analysis of Prior SAHC users

To explore the impact of prior experiences with contraceptive methods on current preferences, we examined these associations for all women who reported any previous lifetime SAHC use (n = 205), including both those who elected SAHC and LARC at the index visit. While 49.7% of women reported dissatisfaction with prior SAHC, this was not predictive of LARC uptake (*p* = 0.97). The majority of women experienced menstrual changes (86.6%) or other side effects (41.0%) while using SAHC, however these symptoms were not correlated with LARC uptake over return to short-acting methods (p = 0.95 and p = 0.182, respectively). This remained true even among the subset of women who reported discontinuation of SAHC specifically due to these side effects (menstrual changes p = 0.89; other side effects p = 0.28). Women who discontinued SAHC to become pregnant were more likely to return these methods postpartum (LARC 4.7% vs SAHC 20.0%, *p* = 0.002).

### Multiple logistic regression model of LARC uptake

Age, number of pregnancies, and the significant variables at p<0.10 from the univariate analysis (prior use of SAHC, number of prior methods used, and current use) were included in the final model (Table 2). Only current use of contraception (OR 3.67, 95% CI 1.67–8.04) and age (OR 0.92, 95% CI 0.85–0.98) were significantly correlated with LARC uptake. Prior SAHC (p = 0.46), number of prior methods (p = 0.66), and number of pregnancies (p = 0.050) were not significant in the final adjusted model. Tests of model fit revealed a likelihood ratio chi-square of 24.4 (p<0.05), a Hosmer-Lemeshow goodness of fit test chi-square of 5.95 (p = 0.65), and a pseudo R^2^ of 0.085.

## Discussion

### Main finding: The role of access barriers

According to Guatemalan national data, among the 48.9% of women currently using a modern contraceptive only 6.9% use a LARC method. Here we present data from a family planning program that removed multiple barriers to contraception by providing a full range of methods at no-cost and in remote locations by culturally-competent providers. In this context, a majority (79.2%) of women seeking contraception elected a LARC method (Table 1).

This finding supports the growing body of literature from LMICs to support the hypothesis that access barriers—as opposed to simply patient preference for SAHC—are an important cause of low LARC utilization in low-resource settings [2, 17, 18, 21]. Comparing our findings to high-resource settings, the same may also be true. For example, the Contraceptive CHOICE project conducted with a vastly different population in St. Louis, Missouri [31] found that 67% of low-income participants selected a LARC method over short-acting alternatives when offered via health access points at no cost.

### Comparison to existing research on prior methods experience and preference

To inform future efforts to target provision of implants and IUDs to the populations likely to desire in these methods, we explored demographic and clinical characteristics of our population that correlated with LARC uptake. Prior research in both high-income countries and LMICs on LARC uptake has explored demographic features, finding higher LARC use among older women and those with higher parity or education [12, 13, 32, 33]. In line with these studies, we also found that older age correlated with uptake in the multiple regression model (OR 0.92, 95% CI 0.85–0.98), although number of pregnancies was just outside of statistical significance (p = 0.050 at cutoff p < 0.05).

In addition, two studies from LMICs have examined whether prior contraceptive experience with SAHC influences decisions about LARCs, but with conflicting results [11, 13]. In this study, prior use of SAHC did not correlate with LARC uptake when controlling for other factors (OR 1.51, 95% CI 0.51–4.47). Furthermore, while women commonly cited unwanted side effects from SAHC, these negative experiences did not correlate with increased LARC uptake. One potential, albeit speculative, explanation is the fact that nurses in the program examined here provided in-depth counseling to all women. This may have alleviated fears from users experiencing benign side effects of SAHC, and encouraged those disposed to a SAHC to elect the method despite their prior experience [34]. It is also possible that subconscious motivations, such as internal inconsistency about actual desire to avoid a pregnancy, may steer some women toward less efficacious SAHC [35].

Studies in other LMICs have found most implant users initiate post-partum or from contraception non-use [2]. In our study women in these two categories were more likely to pick SAHC over implants. Furthermore, new implant users were more likely to directly transition from use of SAHC. Taken together, these results may indicate that women with more contraception experience are switching to LARCs not because of dissatisfaction with SAHC, but rather due to the desire for a more efficacious method when such becomes available [36], with SAHC use still remain a common strategy postpartum and for many never-users.

### Limitations

Our study has important limitations. Most importantly, it involves a highly selected population of women seeking contraception at one institution in rural Guatemala. Since current medical records at MHA do not document discussion of contraception options for women who choose not to select a method, the population here may preferentially include many early adopters or individuals specifically seeking a LARC and may therefore over-estimate LARC uptake rates. For the same reason, the data may underestimate SAHC use because these methods may be more easily obtained from sources outside MHA.

At the same time, the MHA communities included here are isolated, with little alternative access to methods either through public health posts or pharmacies. Furthermore, the baseline demographic and clinical characteristics (including, age, number of pregnancies and living children, baseline use of modern contraceptive methods, and types of prior contraceptive method choices, Table 1) of our sample are similar to the most recent 2015 Demographic Health Survey from Guatemala (6). Nevertheless, the results presented here must be considered exploratory and will need further study to confirm their generalizability.

Furthermore, as a retrospective chart review, we could not examine all possible demographic predictors—such as relationship dynamics [37]—or evaluate patient knowledge. As a result, the overall explanatory power of our regression model is modest (pseudo R^2^ 0.085). Because most regression models for predicting use of long-acting contraception have not published metrics of fit, we are unable to directly compared performance [11–13, 38]. Finally, demographic surveys and previous studies have shown the population represented here to be primarily of Maya descent [27, 28, 39], however we did not collect this as a person-level variable. Lastly, our results do not inform decision-making regarding sterilization or use of natural family planning methods, which were not offered.

### Future directions

Given that mobile family planning programs such as the one presented here appear to have a positive impact on LARC uptake [16] rigorous implementation science should be used to understand the aspects of design and execution are integral to program success, such as frequency of visits and model for patient follow-up. Furthermore, while interest in implants among our cohort was high, only 2.2% of LARC users chose a copper IUD. Future work should investigate uptakes rates of progesterone IUDs using a similar model to overcome barriers, as well as test approaches to copper IUD demand generation. Of note, we are currently conducting qualitative interviews with LARC and SAHC users to explore and confirm our quantitative findings shared here.

More research is also needed to clarify how prior LARC experience influences subsequent decision-making about contraception, as baseline rates of LARC utilization were too small in our cohort to draw any conclusions. The data presented here also do not inform long-term continuation rates, switching behavior, and satisfaction with method choice, all of which are important issues for programmatic decision-making that we plan to address in future research.
